# BM study: a monocentric prospective observational cohort study on neonatal humoral immunity against COVID-19 secondary to transplacental antibody transfer and breastfeeding

**DOI:** 10.1186/s13052-025-02042-3

**Published:** 2025-07-01

**Authors:** Rosa Perretta, Juan José Borraz Torca, Giuseppina De Luca, Marta Donà, Martina Gasparella, Elisa Rizzato, Nicola Bertazza Partigiani

**Affiliations:** 1 Pediatrics Unit of Arzignano Cazzavillan Hospital, Berica, Arzignano, AULSS8 Italy; 2 Department of Women’s and Children’s Health, Padua University Hospital, University of Padua, Via Nicolò Giustiniani, 2, Padua, 35128 Italy

## Abstract

**Background:**

The COVID-19 pandemic highlighted the vulnerability of pregnant women and newborns, emphasizing the importance of vaccination during pregnancy to protect mothers and their infants. Maternal vaccination generates robust anti-SARS-CoV-2 IgG antibodies, transferred to the fetus through the placenta, providing neonatal protection. Breastfeeding also transfers maternal antibodies (IgA and IgG), potentially enhancing mucosal immunity. This study aimed to evaluate neonatal antibody kinetics following maternal COVID-19 vaccination, comparing vaccinated naïve mothers to those previously infected.

**Methods:**

A monocentric prospective observational cohort study was conducted between July 2021 and July 2022, enrolling 44 mother-infant pairs at the Pediatrics Unit of Arzignano Cazzavillan Hospital. Eligible participants included mothers vaccinated during pregnancy (third trimester), either naïve or previously infected with SARS-CoV-2. Blood and breast milk samples were collected at birth and periodically up to six months postpartum. Neonatal cord blood and subsequent samples were analyzed for anti-SARS-CoV-2 spike (S1) IgG and IgA antibodies. Statistical analyses involved parametric and non-parametric tests, with significance set at *p* < 0.05.

**Results:**

Out of 44 enrolled pairs, 12 breastfeeding pairs (9 naïve and 3 previously infected mothers) and 3 formula-fed pairs completed the full protocol. At birth, neonates demonstrated significantly higher IgG levels than mothers, especially from naïve mothers, confirming efficient transplacental antibody transfer (*p* < 0.05). Breast milk from previously infected mothers contained significantly higher IgA levels than naïve mothers at all postpartum time points (*p* < 0.05), whereas IgG levels remained stable and similar between groups. Formula-fed infants exhibited a faster decline in serum IgG compared to breastfed infants.

**Conclusions:**

Maternal vaccination during pregnancy induced robust transplacental IgG transfer, providing neonatal protection from birth. Breastfeeding significantly maintained neonatal IgG levels and contributed additional IgA-mediated mucosal protection, particularly following maternal infection. Formula-fed infants experienced a more rapid antibody decline. Maternal COVID-19 vaccination effectively transfers protective antibodies transplacental and through breastfeeding, suggesting its importance in prenatal care strategies.

## Background

The COVID-19 pandemic has profoundly impacted global public health, with significant risks for vulnerable populations, including pregnant women and neonates. Pregnancy is associated with physiological immunomodulation, potentially increasing susceptibility to severe SARS-CoV-2 infection and adverse pregnancy outcomes such as preterm birth, preeclampsia, and neonatal morbidity [1]. Vaccination during pregnancy emerged as a crucial preventive strategy to reduce maternal morbidity and confer protection to the newborn through passive immunity.

It was described that maternal vaccination against SARS-CoV-2 results in the robust generation of specific maternal antibodies, primarily immunoglobulin G (IgG), which are efficiently transmitted to the fetus via the placenta. Such transplacental transfer shown to provide protective levels of antibodies to neonates at birth, potentially reducing the early risk of infection [1–2]. However, breastfeeding is another critical route by which maternal antibodies, especially immunoglobulin A (IgA), confer mucosal protection to newborns, complementing systemic IgG-based immunity [2–3]. Other studies confirmed the presence of anti-SARS-CoV-2-specific antibodies (IgA and IgG) in breast milk following maternal vaccination [4]. Notably, a prospective cohort study conducted in Israel demonstrated that breastfeeding mothers vaccinated with the Pfizer-BioNTech vaccine developed detectable levels of anti-SARS-CoV-2 IgA and IgG antibodies in breast milk as early as 21 days after vaccination [5]. These antibodies present a significant neutralizing capacities, suggesting a critical role in neonatal mucosal protection against SARS-CoV-2 infection. However, the longevity and dynamics of these passively transferred antibodies, particularly comparing mothers with prior natural SARS-CoV-2 infection and those naïve prior to vaccination, remain areas of active research. Natural infection induces strong mucosal immunity, primarily mediated by IgA antibodies, which differ qualitatively and quantitatively from those generated through systemic vaccination [6–7]. COVID-19 vaccination strategies, especially for vulnerable populations like pregnant women and infants, understanding the duration, composition, and effectiveness of passively acquired neonatal antibodies becomes essential. In this context, we designed a prospective observational cohort study to evaluate the transfer and persistence of maternal SARS-CoV-2-specific antibodies in neonates over the first six months postpartum. Specifically, our study investigates the antibody dynamics transmitted via transplacental passage and breastfeeding, comparing infants born to mothers naïve at vaccination with those who had previously contracted SARS-CoV-2 infection. This investigation seeks to clarify the relative contributions of vaccination versus natural infection in shaping neonatal passive immunity, potentially informing maternal vaccination recommendations and breastfeeding practices during pandemics.

## Methods

The BM study is a monocentric prospective observational cohort study conducted in the Pediatrics Unit of Arzignano between July 2021 and July 2022. The inclusion criteria for the study required neonates to be born to mothers who were either vaccinated for COVID-19 or had contracted a native infection before the last trimester of pregnancy as further described. Moreover, only neonates who were exclusively breastfed or exclusively formula-fed were eligible for participation. The feeding method was chosen antenatally and documented before delivery, based on maternal intention or clinical contraindications to breastfeeding. Conversely, the exclusion criteria ruled out mothers who were neither vaccinated nor had a history of native COVID-19 infection. Newborns receiving complementary feeding were also excluded, as were mothers who declined to participate in the study. The BM study protocol was introduced to expecting to mothers by pediatricians during prenatal preparation courses or by obstetric staff during routine prenatal check-ups at 34 weeks of gestation or immediately postpartum in the maternity ward. Participation in the study was offered to all mothers during the study period to minimize selection bias. Written informed consent was obtained. At delivery (T0), maternal blood samples were collected to measure anti-nucleocapsid (NCP) IgG levels, which were used as a marker to evaluate whether the mother contracted a previous native COVID-19 infection, as the evidence of NCP IgG is exclusive to individuals who had contracted a native COVID-19 infection [8].

The primary aim of the study was to determine whether exclusive breastfeeding is associated with higher anti-Spike IgG or IgA antibody levels in infants compared to those fed with formula milk. The secondary aim was to explore the variation in anti-Spike IgG or IgA antibody levels in infants based on the mother’s COVID-19 immunization status, differentiating between prior infection and vaccination. This classification was based on clinical history and the presence of NCP IgG antibodies.

### Baseline population data

The collected baseline maternal data included maternal age, previous SARS-CoV-2 infection status (confirmed via serology), vaccination timing, and any relevant pregnancy-associated medical conditions such as gestational diabetes, preeclampsia, or infectious complications. Neonatal data gathered at birth included gestational age (weeks), kind of delivery (eutocic, cesarean, or operative), anthropometric parameters such as birth weight (grams), length (cm), and head circumference (cm). Furthermore, the clinical status of newborns was evaluated through the Apgar score at 5 min postpartum, the requirement for neonatal resuscitation procedures, and any admission to the neonatal intensive care unit (NICU). Additional neonatal variables included infant sex, birth order (primiparity or multiparity), the presentation at birth (normal or abnormal) and the presence of newborn malformations.

Data collection was performed immediately postpartum, with neonatal cord blood samples analyzed for IgG and IgA anti-spike antibodies. Maternal blood samples at delivery were simultaneously evaluated for anti-nucleocapsid and anti-spike IgG antibodies, establishing the baseline immune status of each participant pair. All baseline data were recorded and stored securely using electronic spreadsheets designed for the study, enabling accurate tracking and data integrity throughout the duration of the research.

### Sample collection

**Fig. 1:**
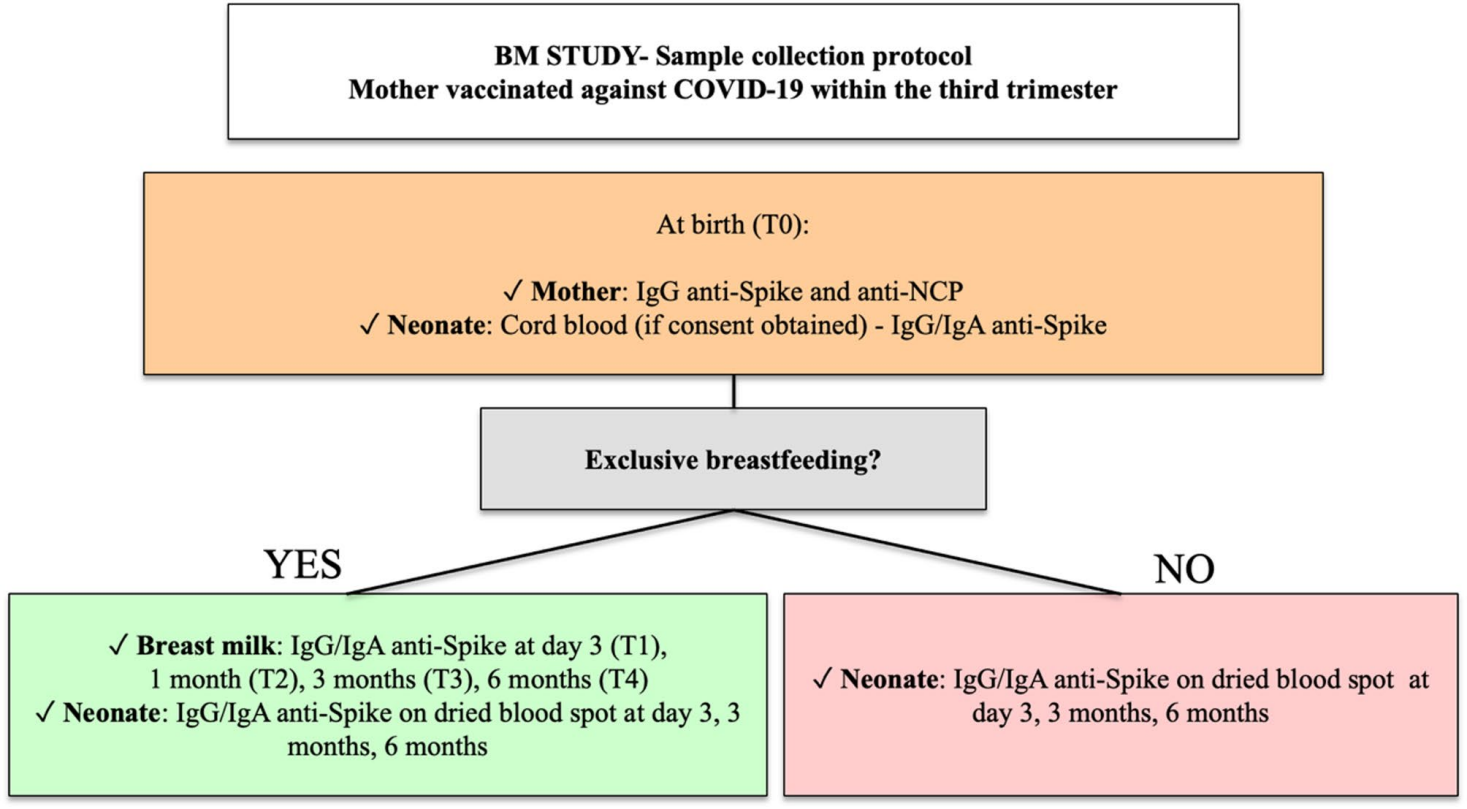
Flow chart of sample collection and follow-up in the BM Study. NCP: anti-nucleocapsid

The scheduled sample collection included neonatal blood samples taken at various time points to measure anti-NCP, anti-Spike IgG and IgA levels. At birth, cord blood samples were collected if consent was obtained prior to delivery (T0). Additionally, heel prick samples and breast milk samples were collected on day 3 of life/post-partum (T1) during routine metabolic screening, as well as at three months (T2) and six months (T3). The following sections will detail the methods of sample collection and analysis, as presented in Fig. 1.

### Neonatal blood collection

Samples for measuring anti-NCP, anti-Spike IgG and IgA were collected on dried blood spot (DBS) cards (ZV 9701 − 0101, Euroimmun AG). In particular, five drops of capillary blood were collected via heel prick, deposited on DBS cards, allowed to air dry at room temperature for at least 30 min, and stored at -20 °C until further analysis. Cord blood was not collected if consent was obtained postpartum. Samples were analyzed via ELISA to quantify IgG antibodies against the NCP (anti-SARS-CoV-2 NCP ELISA, IgG, EI 2606-9601-2 G, Euroimmun AG, Germany) and the S1 domain of the Spike protein of SARS-CoV-2 (Anti-SARS-CoV-2 QuantiVac ELISA, IgG, EI2606-9601-10 G, Euroimmun AG, Germany) following manufacturer instructions. In particular, DBS cards were diluted in 250 µL of sample buffer and incubated for 1 h at 37 °C. The resulting supernatant was used for semi-quantitative and quantitative immunoenzymatic analysis. NCP test results were classified as negative (< 0.8), borderline (≥ 0.8 to < 1.1), or positive (≥ 1.1). A positive result indicated infection within one month prior to testing. Anti-S1 test results were classified as negative (< 25.6 BAU/mL), borderline (≥ 25.6 to < 35.2 BAU/mL), or positive (≥ 35.2 BAU/mL).

### Breast milk collection

Anti-Spike IgG and IgA levels were measured in breast milk at 2 and 4 weeks, as well as at 3 and 6 months postpartum. Breast milk samples (5 mL) were collected in Falcon tubes, centrifuged, and stored at -20 °C in the laboratory until further analysis. Breast milk samples were collected at T0 (three days), T1 (one month), T2 (three months), and T3 (six months). Before analysis, samples were thawed at room temperature, centrifuged at 3,000 rpm for 10 min, and analyzed via ELISA for IgA (Anti-SARS-CoV-2 IgA, EI 2606-9601-A, Euroimmun AG, Germany) and IgG antibodies targeting the S1 domain of the Spike protein (Anti-SARS-CoV-2 QuantiVac ELISA, IgG, EI 2606-9601-10 G, Euroimmun AG, Germany). Anti-IgA test results were classified as negative (< 0.8), borderline (≥ 0.8 to < 1.1), or positive. Quantitative anti-S1 tests were interpreted as described above.

### Statistical analysis

Data were analyzed using Excel and GraphPad Prism 9 software. Continuous variables were summarized using means and standard deviations (SD) or medians and interquartile ranges (IQR) according to their distribution, assessed through Shapiro-Wilk tests. Categorical variables were presented as frequencies and percentages. Comparisons of continuous variables between two groups (naïve vaccinated vs. previously infected mothers, breastfed vs. formula-fed infants) were performed using Student’s t-test for normally distributed data or the Mann-Whitney U test for non-parametric distributions. Paired comparisons, where applicable, were evaluated through paired t-tests or Wilcoxon signed-rank tests. The levels of antibodies (IgA and IgG) measured in maternal and neonatal samples over time were analyzed using repeated-measures ANOVA or Friedman tests, depending on data distribution. A *p*-value of less than 0.05 was considered statistically significant. Due to the limited sample size, exploratory rather than confirmatory statistical approaches were emphasized, and findings should be interpreted cautiously. A web-based statistical tool was also used (https://www.socscistatistics.com/tests).

## Results

### Population

**Fig. 2:**
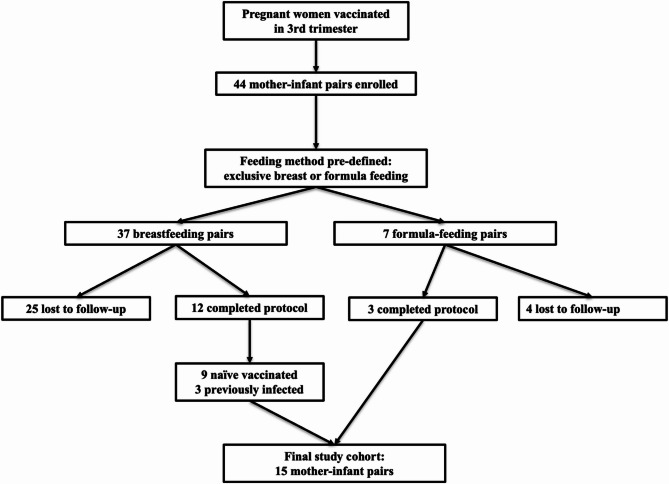
Flow chart of participant enrollment in the BM Study

A total of 44 mother-infant pairs were enrolled, all mothers having received SARS-CoV-2 vaccination during pregnancy. Of these, 37 mothers were breastfeeding, and 7 mothers chose artificial milk feeding. Out of the breastfeeding group, 25 pairs (67.6%) did not comply fully with the sampling schedule and were thus excluded from further analysis. Consequently, 12 breastfeeding pairs (32.4%) completed the entire protocol timeline and were classified as follows: 9 pairs with vaccinated naïve mothers, and 3 pairs with vaccinated previously infected mothers (confirmed by positivity to anti-nucleocapsid antibodies, NCP-Ab, at baseline T0). Regarding artificial milk-fed infants, complete datasets were available for 3 out of the initial 7 pairs (42.9%), all negative for NCP-Ab at T0. The flow-chart of population selection is presented in Fig. 2.

The neonatal population showed a predominance of male infants, accounting for 73% of cases. All newborns were delivered at term, with a median gestational age of 39 weeks (interquartile range: 39–41). The median birth weight was 3310 g (IQR: 3075–3540), and the Apgar score at five minutes was uniformly optimal, with a median value of 10 (IQR: 10–10). Only 20% of the infants were firstborns, and none required admission to the neonatal intensive care unit (NICU).

Regarding the mode of delivery, the majority of neonates (80%) were delivered vaginally, while 20% were born via cesarean section. With respect to maternal comorbidities during pregnancy, 40% of mothers had gestational diabetes, 7% experienced urinary tract infections, and 7% had threatened miscarriage, while 46% of mothers had no reported complications. As per the study design, 80% of infants received exclusive breastfeeding during follow-up, in line with antenatal maternal intention.

### Main results

**Fig. 3:**
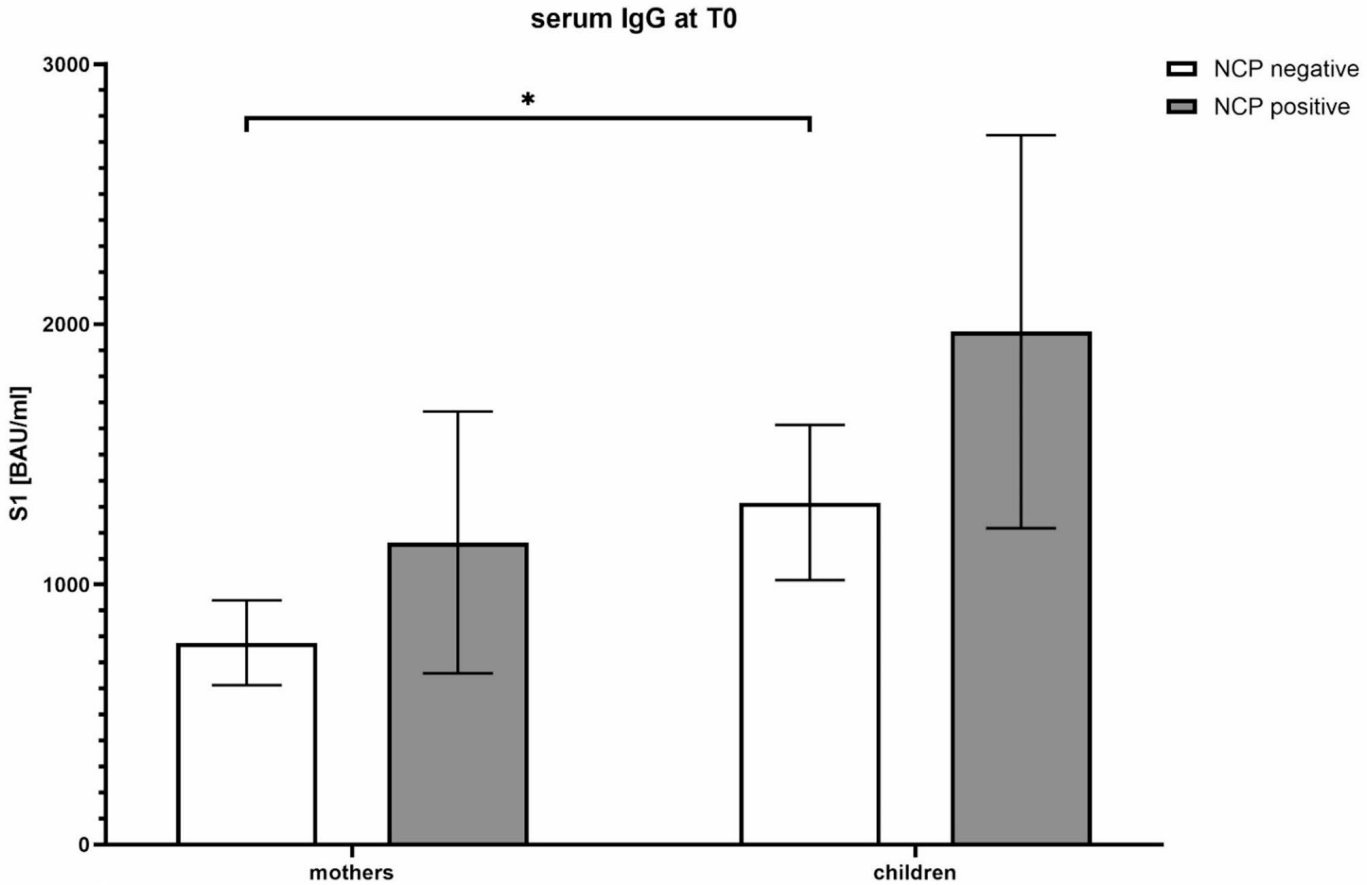
Relationship between anti-spike IgG S1 antibody levels in vaccinated mothers (NCP-negative) or previously infected mothers (NCP-positive) and their neonates at 3 days of life. NCP: anti-nucleocapsid

At T0, maternal serum IgG levels directed against the SARS-CoV-2 spike (S1 IgG) were not statistically different between previously infected (1161.35 ± 259.69 BAU/ml) and naïve vaccinated mothers (776.52 ± 46.12 BAU/ml; *p* > 0.05). However, neonates demonstrated significantly higher IgG levels compared to their mothers, particularly within the naïve mother-infant pairs (*p* < 0.05) (Fig. 3).

**Fig. 4:**
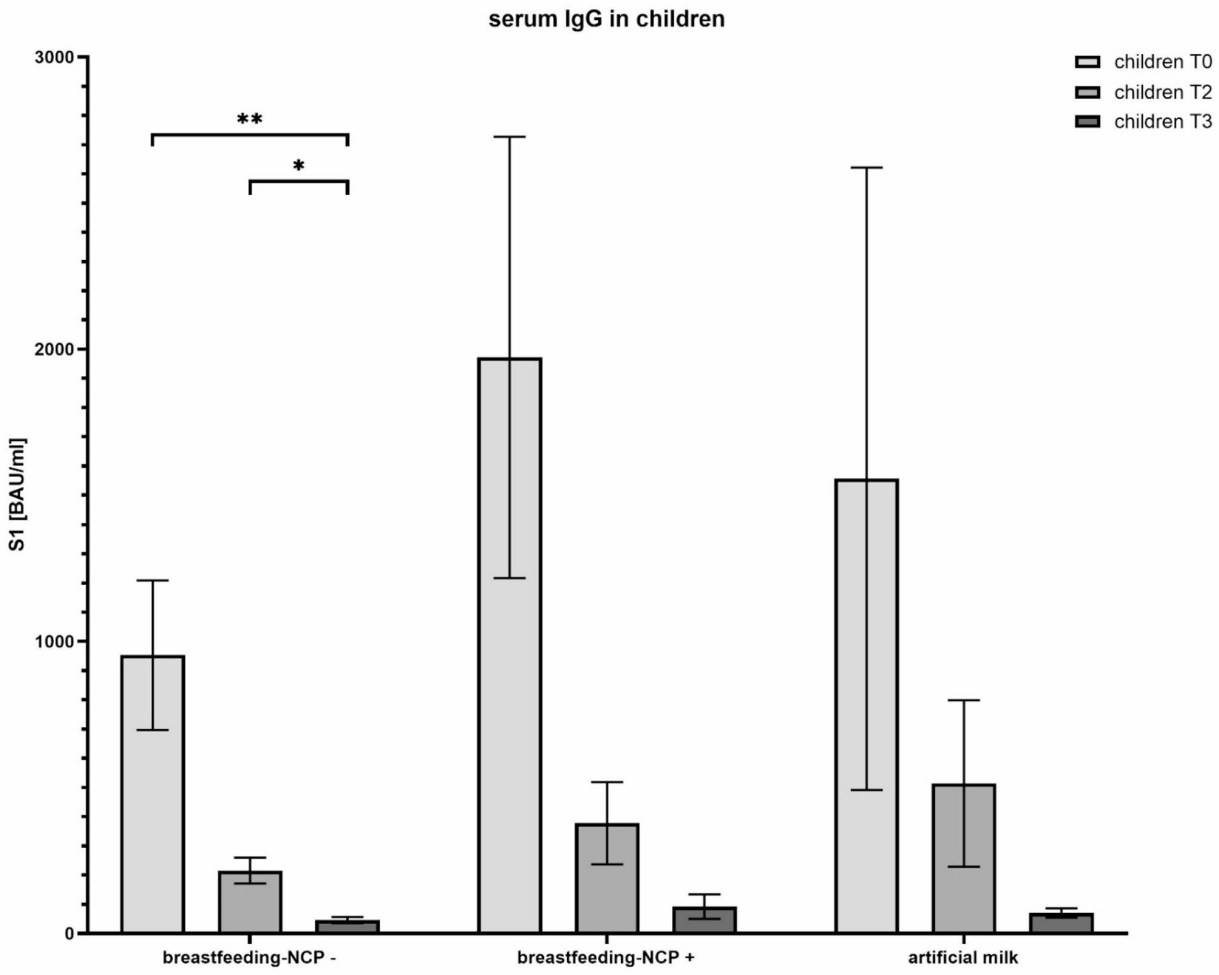
Relationship between neonatal anti-spike IgG S1 antibody levels at 3 days (T0), 3 months (T2) and 6 months of life (T3). [*p* value < 0,05=*; <0,005=**]; NCP: anti-nucleocapsid

Analysis of breast milk samples collected at defined intervals (3 days, 2–4 weeks, 3 months, and 6 months postpartum) revealed significantly higher IgA concentrations in previously infected vaccinated mothers compared to naïve mothers at each time-point (*p* < 0.05). In infected mothers, IgA concentrations decreased progressively from birth (T0: 269.72 ± 85.79 BAU/ml) to 6 months postpartum (T3: 172.33 ± 74.30 BAU/ml). In contrast, naïve mothers presented relatively low and stable IgA levels (T0: 29.69 ± 18.98 BAU/ml; T1: 27.56 ± 19.53 BAU/ml) (Fig. 4). Milk IgG levels did not differ significantly between infected and naïve groups at any time point, although they inclined to remain consistently elevated through the six-month observation period (T3 IgG: naïve group, 137.54 ± 12.88 BAU/ml; infected group: 204.77 ± 26.47 BAU/ml; *p* > 0.05). Particularly, IgG levels were overall higher than IgA in the breast milk of naïve mothers (*p* < 0.05) (Fig. 4).

**Fig. 5:**
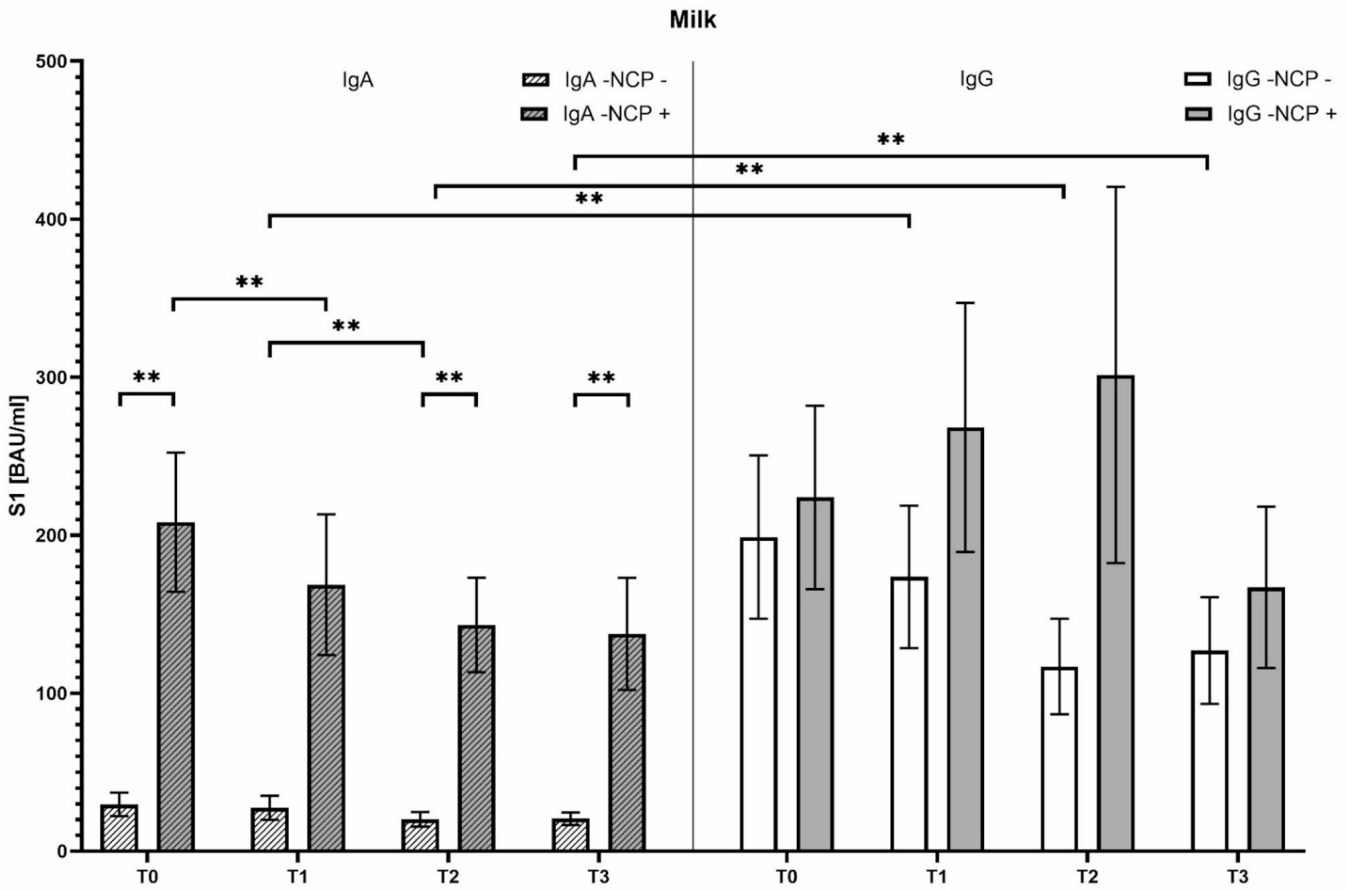
Evaluation of the temporal trend of anti-spike IgA and IgG antibody concentrations in breast milk over time (up to six months postpartum). [pvalue < 0,05=*; <0,005=**]; NCP: anti-nucleocapsid

Finally, serum IgG antibody levels in infants fed with breast milk (both naïve and infected groups) and formula milk-fed infants decreased over the follow-up period. Despite this general decline, a statistically significant reduction was observed only within infants from the naïve breastfeeding group. Interestingly, formula-fed infants experienced a notably faster reduction of serum IgG concentrations, reaching lower levels at six months (70.26 ± 24.55 BAU/ml) (Fig. 5). Nonetheless, due to limited sample size per group, these observations should be interpreted cautiously.

### Discussion

This prospective, monocentric observational study performed in a small Pediatric Department aimed to evaluate the passive immunity against SARS-CoV-2 conferred to neonates by maternal vaccination during pregnancy, particularly focusing on the comparative roles of transplacental transfer (IgG) and breastfeeding (IgA and IgG). Despite the relatively small sample size, our data reinforce the protective effect of maternal immunization and provide insight into the differential immune contributions of previously infected versus naïve mothers. Our findings confirm robust transplacental IgG transfer following maternal SARS-CoV-2 vaccination, leading to elevated antibody titers in neonates at birth. Interestingly, neonates born to naïve vaccinated mothers often showed significantly higher IgG levels than their mothers, underscoring the efficiency of active transport across the placenta. This observation aligns with previous reports indicating that the neonatal Fc receptor (FcRn)-mediated mechanism can enhance neonatal IgG titers relative to maternal levels [1, 2]. In line with previous findings, this supports the hypothesis that IgG transplacental transfer confers passive neonatal immunity even in the absence of maternal symptoms or neonatal infection [10, 11, 12, 13]. Furthermore, we did not detect statistically significant differences in maternal IgG titers at delivery between previously infected mothers and naïve mothers, suggesting that vaccination alone elicits substantial antibody responses. These results support current recommendations advocating vaccination during pregnancy to confer early protection to newborns [13]. This study did not evaluate anti-nucleocapsid IgM or perform molecular testing for SARS-CoV-2 RNA in neonatal samples; therefore, vertical transmission could not be assessed. However, previous studies. report no detection of viral RNA in breast milk or neonatal nasopharyngeal swabs, and only rare detection of IgM in cord blood, suggesting that vertical transmission is an uncommon event [12, 15, 16, 17, 18].

Our study also investigated antibody levels in breast milk, highlighting distinct IgA and IgG patterns between previously infected and naïve vaccinated mothers. Consistent with other reports, previously infected mothers exhibited significantly higher IgA concentrations [3, 6], likely reflecting a robust mucosal immune response triggered by natural infection. Although these IgA titers declined over the postpartum period, they remained detectably elevated for up to six months. Conversely, naïve vaccinated mothers produced lower but relatively stable IgA levels. Notably, both groups maintained measurable IgG concentrations in breast milk, supporting that vaccination predominantly elicits systemic rather than mucosal immunity [2].

In parallel with breast milk findings, serum IgG levels in neonates decreased over time in all groups, yet the decline was more pronounced in formula-fed infants. Although the statistical significance was limited by the small sample size, this trend suggests that breastfeeding may help sustain passively acquired neonatal IgG titers for a longer period. Such an observation is clinically relevant, as it reinforces the potential additive role of breast milk-derived IgG and IgA in bolstering neonatal protection during the first months of life [4, 18].

Two main elements highlight this study. First, the high IgG levels observed in neonates born to mothers with no history of natural infection underscore that vaccination alone is sufficient to confer substantial passive immunity. This finding is particularly important for reinforcing vaccine uptake in pregnant individuals who have not experienced prior infection. Second, the extended follow-up (six months postpartum) offers insights into the durability of both IgA and IgG in breast milk, highlighting a continued, albeit gradually diminishing, supply of maternal antibodies. From a public health perspective, our data support the dual recommendation of maternal vaccination, regardless of prior infection status, and the promotion of breastfeeding to enhance neonatal immune defense during a possible critical window of development.

This study presents many limitations which should be acknowledged. First, the sample size is small, and the subgroups, particularly those involving previously infected mothers, are very limited in number, reducing statistical power. Second, the follow-up attrition rate among breastfeeding pairs was high (67.6%), which may have introduced selection bias and reduced the robustness of longitudinal analyses. Third, we did not assess the neutralization capacity of the detected antibodies, which limits the interpretation of their actual protective potential. Finally, the monocentric design and relatively homogeneous population may affect the generalizability of the findings.

Despite these limitations, our study contributes real-world data supporting the immunological benefits of maternal vaccination and breastfeeding. Larger, multicenter studies are needed to confirm these findings, explore the neutralizing capacity of milk-derived IgA and IgG, and assess the clinical implications for infection rates in neonates.

## Conclusion

In conclusion, this study reinforces the importance of maternal SARS-CoV-2 vaccination during pregnancy, irrespective of previous infection status. Vaccination effectively stimulates a significant maternal IgG response, transmitted through transplacental transfer to neonates at birth, and is further maintained by breastfeeding. Given the ongoing evolution of the COVID-19 pandemic, maternal vaccination represents a valuable strategy for neonatal protection and should be encouraged as part of routine prenatal care.

## Data Availability

The datasets are not publicly available due to privacy and ethical restrictions. However, they are available from the corresponding author on reasonable request and with permission from the Ethics Committee, if applicable.
